# History of a Case of Hydrocephalus Chronicus, in Which Paracentesis Capitis Was Performed

**Published:** 1824-02

**Authors:** Robert Brown

**Affiliations:** Surgeon, Preston; Fellow of the Royal College of Surgeons, &c. London.


Art. III.—*History of a Case of Hydrocephalus Chronicus, in which Paracentesis Capitis was performed.* By Mr. Robert Brown, Surgeon, Preston; Fellow of the Royal College of Surgeons, &c. London.

The division which has been made of hydrocephalus into acutus and chronicus, is, I think, very judicious and practical, and furnishes us with a correct notion of the pathology of these varieties of the same disease,—the one variety truly sthenic, the other asthenic. The former is, from the commencement, to be considered as a genuine instance of idiopathic phrenitis, and the indication of treatment to be such as will lessen the momentum of the circulation within the head. Such objects are to be fulfilled by the rigorous adoption of those measures denominated antiphlogistic, and the free use of topical evacuants. By an early and liberal employment of these, few cases of hydrocephalus acutus, if seen at the onset of the disease, will fail to be arrested, and prevent the deposition of that fluid, which constitutes an important, indeed principal, feature of the complaint.

I pretend not to offer any remarks on the difficulty of distinguishing the *acutus* from those diseases which are occasionally found to simulate it: attention to the symptoms present during an attack, coupled with the history of the little sufferer, will generally, I am persuaded, lead to a discrimination of the true nature of the ailment. The object of my present communication is the history of a case of hydrocephalus chronicus, in which the operation of paracentesis capitis was resorted to: but, although in this case it was unsuccessful, I am led to request its publication, which will, when considered in conjunction with the already-published cases, afford strong grounds for hope that, could the prejudices of the relatives be removed, I am almost so sanguine as to think that we have at length acquired the much wished-for desideratum for the cure of this hitherto-deemed incurable disease. Even in the most hopeless cases, it may, I think, with great propriety, be proposed as a dernier resource for the palliation of those disagreeable symptoms of a disease, which the carelessness of friends have suffered to proceed to such a length, and when, under such unfavourable circumstances, the employment of the ordinary means would be both futile and useless. The removal of so much fluid as is usually present in the chronic form of this disease, by the most vigorous activity of the absorbent system, aided by the use of the other auxiliary means that have been proposed, is not ever likely to be realized in practice.

Paracentesis capitis, in the chronic form of hydrocephalus, holds out great hopes of eventual success, provided the operation be early employed,—immediately a sense of fluctuation becomes perceptible; and repeated at short intervals, so as gradually to remove the degree of distention, and prevent the disorganization of structure, which is apt to occur from the accumulated bulk of fluid. The great extent of the disease, and the degree of mischief which the brain will suffer with apparent impunity, regarding the sensorial functions, is truly amazing, and almost sufficient to make us discredit the appearances which post-mortem inspections afford us.

The seeming novelty of the practice of puncturing the head, and the want of that comparative ratio of success usually attendant on other surgical operations, is apt to raise the fears of the timid friends, and who refuse its adoption in consequence of some foolish prepossession of its inefficacy, or perhaps through the mischievous interference or persuasion of some intrusive and busy neighbour.

The want of success in the accompanying case was owing, I think, to these circumstances: for, after the first operation, the child was, as will be seen by the report, most surprisingly benefitted; but the time which elapsed between the first and subsequent operations, in consequence of the refractory conduct of the mother, precluded that chance of success which the first operation led us to anticipate.

Robert Cousin's female child, ætatis five months, a twin, has been about three weeks indisposed: the mother says it has been very cross during this time, almost constantly either crying or screaming. She has, for a long time back, almost from birth, been in the habit of giving the child "a little of Godfrey's cordial," (a panacea, with the lower orders, of universal application, for the cure of all diseases of children, the indiscriminate use of which is highly pernicious, from the quantity of opium it contains; and, most probably, it is one of the most prolific exciting causes of infantile disorder.) About a week back it had diarrhœa. At the present time (September 16th, 1823, the first time I have seen it,) it is perfectly quiet: eyelids half closed; pupils immovable to impressions; head very large, and remains constantly upon the side on which it is placed. The mother imagined the head to be larger, for the first time, this morning, whilst dressing. The fontanels enlarged, and the integuments much distended, with a sense of fluctuation perceptible on pressure.

Emplastrum lyttæ nuchæ imponendum.

℞. Unguenti Hydrargyri Fortioris, ℈j. Illinatur Axilla 4ta. vel 6ta. q. q. horâ.

℞. Pulveris Digitalis, gr. vi.
Hydrarg. Submuriat. ℈j.
Mucil. G. Acaciæ, q. s. ft. et divide in pilulas x. dequib capiat j. 3tia omni horâ.

17th.—Blister has not risen. Pupils act somewhat more readily; the child has been very cross and fretful all night; bowels freely open; discharges mixt with skins and slime; the integuments of the head apparently more distended. After a due consideration of the several circumstances of the case, and the very improbable chance of relief being afforded from the use of medicine, having candidly informed the friends of the inefficacy and impropriety of trusting to it alone, I ventured to solicit their permission to remove the water by puncture. The proposal met with great opposition; various pretexts were made as reasons for their objections. After a long time spent in persuasion, consent was obtained. With the assistance of my friend, Mr. C. J. Greenwood, house-surgeon to the Preston Dispensary, who obligingly afforded me his aid in this and all the succeeding occasions, I performed the operation in the following manner:—The child being placed with its head firmly secured, and in a pending position, upon the lap of a woman, I made an incision with a lancet through the integuments at the frontal angle of the right parietal bone, through which external wound I carefully introduced the trocar, after withdrawing the stilet. f. ℥ix. and ʒiij. of a clear lemon-coloured fluid flowed in an uninterrupted stream by the cannula. After the evacuation of the fluid, a bandage was applied, tolerably tight, round the head.

Vespere.—The child has been, since the operation, more alive to external impressions. On waving the hand before its face, the eyelids closed, and the levatores palpebrarum superiores and the corrugatores superficiales muscles were thrown into strong action. The muscular motion of the body seems much more under the influence of volition. Bowels are stated to be very open; urinary discharge in good quantity. On strictly questioning the mother, I find the ointment not to have been used, from the unnecessary fear, instilled into her previously-prejudiced mind by her mischievous neighbours, of its exciting a salivation: she promises now to use it. A little hiccup has affected the child all day.—Persistat in usu omnium medicamentorum.

18th.—Pulse 170; hiccup gone; bowels open; the child lively.

19th.—Pulse 154. Bowels open, and fæces described as of a green colour; eyes stronger, and it seems to possess the power of directing them to any object. On the mother's talking to it, it appears to answer her attentions in the same significant way as young children frequently do. Often yawns, and at times takes the breast voraciously.

20th.—The mother, this morning, states that the child had last night a sort of convulsive fit, which held it some time. The right half of the body, she imagines, has no use in it; has a frequent twitching of the left leg towards the belly. Pulse 160; bowels open; a little cough is sometimes present.—Ointment to be continued: having vomited the pills lately, have directed their use for the present to be suspended.

21st.—The child is quite playful; motion of the limbs free; paralytic state of the right side gone; eyes can be steadily directed to any object, and susceptible to the slightest external impressions; pulse 165; bowels open, and fæces of a yellow colour; urinary discharge copious; cough more troublesome,

℞. Oxymellis Scillæ, f. ℥iij.

Spiritus Ætheris Nitrici, f. ʒij.

Træ. Camphoræ comp. f. ʒij.

Træ. Digitalis, ♏ xxx.

Aquæ Menthæ Pip. f. ℥iij. fiat mistura sumat cochl. parv. ij. tertia omni horâ.

23d.—Pulse 150. Bowels open, fæces of a green colour; urine plentiful; strabismus occasionally affects the left eye. Mother complains that its nights are spent in a very restless way. It frequently moans; fontanels a little more puffy, with a sense of fluctuation. Roller re-applied, and the medicines directed to be continued.

24th.—Has been very restless all night; strabismus sometimes visible at this visit in both eyes; bowels free; pulse but little varied; the vertex much more prominent, and fluctuation more distinct, than yesterday. I proposed a repetition of the operation; but, although decided relief was obtained by the former operation, the mother pertinaciously refused even to listen to our proposal, stating she had no faith in any thing that could be done, and steadily objected to any further attempt being made: she should rather, she said, wish the child to die, than put it (as she foolishly thinks) to more punishment.—A large quantity of the ointment ordered to be rubbed in.

26th.—Pulse 156, and stronger; bowels very open, fæces green, and mixt with skins; cough gone. Mother still complains how ill the child rests in the night. The eyes appear to-day free from strabismus; hectic flushes have appeared several times upon the cheeks both yesterday and to-day, with partial sweats upon the head and face.—Ointment continued, and mixture discontinued.

29th.—Child this morning quite playful, and, when I entered the house, the mother was tossing it about. Has had no strabismus, no hectic flushes, &c. since last report. The integuments covering the fontanels, on taking off the roller, became flaccid, without any sense of fluid. Pulse 157; bowels open.

October 1st.—Child very cross, and has often its fingers in its mouth, as though it was suffering in part from dentition. Pulse 150; bowels very loose, fæces yellow; urinary discharge plentiful.

4th.—The mother informs me that the child yesterday had great twitchings of its hands and legs. Eyes, I observe, are occasionally, this morning, affected with strabismus; is very cross; cough has again troubled it; fluctuation more distinct at the anterior fontanel. I have this morning applied straps of adhesive plaster instead of the roller, which will afford more equable pressure and support. I have discovered that the mother has very imprudently been gossiping with the child in her neighbour's, and also neglected the use of the ointment.

6th.—Child is very cross, and always, when awake, rubbing its gums; diarrhœa profuse; appearance of the head similar to that described in last report.

℞. Misturæ Cretæ, f ℥iv.

Træ. Opii, ♏ x.

Træ. Catechu, f. ʒij. fiat mistura.

Sumat cochl. magnum post singulas sedes liquidas.

8th.—Child out when I called: it is very quiet during the day, but fretful and cross during the night; the head appears to be better supported by the muscles of the neck; fluctuation of water more evident than at last visit; diarrhœa abated; a strophulous eruption covers the body.—Ointment still to be continued; absorbent mixture omitted.

10th.—The head appearing more enlarged, and the accumulation of fluid more obvious, I again strenuously urged the necessity of our resorting to the operation; but no arguments will prevail with the stubborn mother to allow us the required permission, in consequence of which I shall suspend my attendance, until I am solicited to renew it.

16th.—The child's father waited upon me last night, and apologised for the obstinacy of his wife, in not allowing me to act towards the child as I deemed fit. He requested me to call this morning; and, if I thought the operation held out any prospect of benefiting the child, he gave me full discretionary power to act accordingly. The child is greatly altered in appearance since my last visit: it lays in a perfectly comatose state, and, to use the expression of the friends, "is blind with its eyes open." The eyes devoid of sensibility, and apparently sunk within the orbits; integuments of the cranium exceedingly tense; the measurement of the head as follows:—in circumference, 18½ inches; from ear to ear, 11¾. Bowels stated to be constipated. I again performed the operation in the same manner as before, in the left side, in the anterior fontanel, about mid-distance from the superior longitudinal sinus; but nothing except blood escaped by the cannula. I made another puncture about an inch distant from the first externally. Blood also flowed at first from this orifice, and several pieces of brain were discharged; by varying the position of the cannula, water of a sero-sanguineous colour, f. ℥ixss. escaped. Firm pressure was applied to the head during its discharge. After the operation the child became very sick, and vomited, and had a large alvine motion: soon afterwards the sickness subsided, and the child became perfectly sensible, and directed its eyes to surrounding objects, and smiled when noticed by the mother. Roller applied.

℞. Hydrargyri Submuriatis, ℈j.

Pulveris Digitalis, gr. iv.

Mucil. G. Acaciæ, q. s. ft. pil. vj. Sumat j. omnia 8ta horâ.

17th.—The child is very fretful; strabismus again present, and head apparently tense. Bowels open.

18th.—This morning the child is quite lethargic; complete paralysis prevails; integuments of the head quite distended; bowels still open. A small quantity of fluid having oozed out of the wound made in the last operation, I chose it again for the purpose of introducing the cannula, and without any difficulty drew away f. ℥vj. ʒij. of a similar coloured fluid as removed on the former occasion. After the operation, the child became much more sensible, and all the previous alarming appearances vanished. Compression during the operation, as before; afterwards, the adhesive straps.

19th.—The child has had a very good night, and this morning is very lively, and its general appearance materially amended. Bowels very open; fæces varying greatly in colour. Ointment and pills to be continued.

20th.—This morning the child has again become comatose: no part of the eyes appear but the conjunctiva. It has a very cadaverous look, the integuments appearing distended. I again introduced the trocar at the frontal angle of the right parietal bone, and drew away, in one continued stream, f. ℥ivss. of blood, which coagulated immediately in the vessel in which it was received. During the operation the child was once or twice very sick, vomited, and had convulsive motions of the body; stertorous breathing, with a distressing moaning noise. I remained with it about half an hour, expecting every moment to witness it expire. In about an hour after the operation, it partook of a small quantity of the breast; and, in the evening, I learnt it had continued all day to manifest the same death-like appearance.

21st.—It has still the same moribund appearance as yesterday. The cranial integuments are again very tense, from (I fear) internal hemorrhage. Bowels open; takes the breast occasionally in a voracious manner; at other times has not the power either to hold the nipple or to swallow.

22d.—A messenger came in great haste this morning to acquaint me "that the child's head had burst, and a great quantity of bloody fluid had come away." On my arrival, I found the fluid to have escaped from the puncture made on the 18th: from appearance, several ounces must have been discharged. I introduced a probe into the orifice, (being the only instrument the mother would permit me to use,) and employed pressure to the sides of the head, by which means I removed f. ℥iiss. of a bloody-tinctured serum. Roller to the head again resorted to. The child, since the loss of the fluid, has experienced an obvious amelioration; vision is again restored, and unattended by strabismus. Child takes the breast, and can support its head tolerably well. Bowels open.—Pills continued, and ointment discontinued.

23d.—The child has recovered most surprisingly; has again become playful, and sensible to surrounding objects, and to the caresses of its mother: it is sometimes stated to be cross. Bowels free; a bloody-coloured fluid continues to ooze from the wound, and parents think as much or more than our last report states has been discharged. I introduced a director with the groove downwards, which facilitated the escape of f. ℥iss. of the same coloured fluid as before. Integuments quite flaccid, and overlap. Roller re-applied.

24th.—Towards the evening of yesterday, the child was seized with a convulsive fit, but it had ceased from about noon to take the breast: the convulsions became more severe till near midnight, when it became perfectly calm. It awoke the mother, about two o'clock this morning, by the rattling noise it made in breathing, and in about half an hour from this time, whilst in an apparent slumber, it quietly expired. With great difficulty I obtained permission to inspect the head. Assisted by the friend who aided me in the operations, and by two medical pupils, to-day, at six o'clock P.M., I made the following hasty

*Dissection.*—The integuments were divided from ear to ear. Each department of the skull was easily separated, owing to the bones being held together by membranes only. Dura mater firmly adhering. Tunica arachnoidea easily raised. Cerebrum very pale, and extremely soft. On separating the hemispheres, a quantity of water escaped, so as to prevent me from making a methodical demonstration of the parts: even the accidental movement of the head occasioned a gush of water to burst up the corpus callosum, and lay open the lateral ventricles. Through the rent in the corpus callosum was observed the foramen of Monro, so enlarged as to admit the extremity of the finger into its orifice. The fornix, from the pulpiness of the brain, could not be distinctly shown: its two anterior crura were, however, very visible. The infundibulum somewhat increased in size. The lateral ventricles greatly enlarged, and their parietes extremely thin; they were distended with a sero-sanguineous coloured fluid. In the posterior cornua of the left lateral ventricle was observed a piece of coagulated blood, about the magnitude of a hazel-nut. The choroid plexus very pale, and covered with a large cluster of small hydatids. From the free communication existing between each ventricle, water was found in all. The quantity of fluid removed by the examination amounted to about a quart.

*Preston; November 11th, 1823.*

